# Ribosome selectivity and nascent chain context in modulating the incorporation of fluorescent non-canonical amino acid into proteins

**DOI:** 10.1038/s41598-022-16932-7

**Published:** 2022-07-27

**Authors:** Michael Thommen, Albena Draycheva, Marina V. Rodnina

**Affiliations:** Department of Physical Biochemistry, Max Planck Institute for Multidisciplinary Sciences, Göttingen, Germany

**Keywords:** Ribosome, Biological fluorescence

## Abstract

Fluorescence reporter groups are important tools to study the structure and dynamics of proteins. Genetic code reprogramming allows for cotranslational incorporation of non-canonical amino acids at any desired position. However, cotranslational incorporation of bulky fluorescence reporter groups is technically challenging and usually inefficient. Here we analyze the bottlenecks for the cotranslational incorporation of NBD-, BodipyFL- and Atto520-labeled Cys-tRNA^Cys^ into a model protein using a reconstituted in-vitro translation system. We show that the modified Cys-tRNA^Cys^ can be rejected during decoding due to the reduced ribosome selectivity for the modified aa-tRNA and the competition with native near-cognate aminoacyl-tRNAs. Accommodation of the modified Cys-tRNA^Cys^ in the A site of the ribosome is also impaired, but can be rescued by one or several Gly residues at the positions −1 to −4 upstream of the incorporation site. The incorporation yield depends on the steric properties of the downstream residue and decreases with the distance from the protein N-terminus to the incorporation site. In addition to the full-length translation product, we find protein fragments corresponding to the truncated N-terminal peptide and the C-terminal fragment starting with a fluorescence-labeled Cys arising from a StopGo-like event due to a defect in peptide bond formation. The results are important for understanding the reasons for inefficient cotranslational protein labeling with bulky reporter groups and for designing new approaches to improve the yield of fluorescence-labeled protein.

## Introduction

Fluorescence probes provide important insights into the structure and dynamics of macromolecules, but the repertoire of potential approaches for site-specific labeling is still limited^1^. In principle, incorporation of non-canonical amino acids (nc-aa) into recombinant proteins allows for residue-specific labeling with fluorescence reporters and methods for engineering such proteins have advanced rapidly in the past years^2–4^. The development of approaches for chemical aminoacylation of pdCpA dinucleotides and ligation to tRNA lacking two nucleotides at the 3′-end by T4 RNA ligase allowed for chemical charging of tRNA with nc-aa and showed that these chimeric nc-aa are incorporated into nascent peptides by the ribosome^5,6^. A general approach for incorporation of nc-aa in vivo became accessible by the expansion of the genetic code with orthogonal aminoacyl-tRNA synthetase–tRNA pairs that do not charge endogenous tRNA and, vice versa*,* are not substrates for the endogenous aminoacyl-tRNA synthetases, respectively^7^. Further progress came with the selection of mutant ribosomes with improved decoding efficiency of amber stop codon^8^ or quadruplet codons^9^ with the help of respective suppressor tRNAs. Ribozyme (Flexizyme)-mediated aminoacylation is an alternative method to prepare nc-aa-tRNA in vitro^10,11^. The combination of chemical and enzymatic aminoacylation methods made it possible to incorporate nc-aa site-specifically and with high yield^12,13^, providing the tools to synthesize designer proteins with desired properties. In the meantime, a large variety of nc-aa that were sterically restricted, modified in the backbone, or carried biophysical probes has been incorporated into proteins^14–17^. In some cases, in particular for *in-vitro* experiments, natural amino acids such as lysine, cysteine or N-terminal methionine can be used for specific cotranslational fluorescence labeling^18–20^, obviating the need to use genetic code expansion tools^21–23^. Many different fluorophores are well tolerated at the N-terminus of the nascent proteins. However, obtaining high cotranslational labeling efficiency at the internal positions remains a challenge and the potential differences in performance between nc-aa-tRNA and canonical aa-tRNA during the steps of decoding, peptide bond formation and translocation on the ribosome remain largely unexplored^24^.

Here we systematically explore the reasons for potentially low incorporation yield of fluorescence dyes into internal position of proteins using *in-vitro* translation approaches and Cys-tRNA as a scaffold for aa labeling. As fluorescence reporters, we used 7-nitrobenz-2-oxa-1,3-diazol-4-yl (NBD) and 4,4-difluoro-5,7-dimethyl-4-bora-3a,4a-diaza-s-indacene (BodipyFL), which were successfully used as fluorescence probes for cotranslational incorporation into proteins^21–23,25–30^. We also used Atto520 as it can be used for single-molecule studies^31–35^. The optimized *in-vitro* translation setup enabled us to identify limiting steps from decoding to peptide bond formation for fluorescent nc-aa-tRNA that were crucially modulated by the nascent chain context and length on the peptidyl-tRNA. We also identified a StopGo-like event resulting in formation of unlabeled truncated N-terminal and fluorescent-labeled C-terminal peptide products, which decreases the labeling yield of the full-length protein but may be developed into a useful tool to produce labeled peptide chains of defined stoichiometry.

## Results

### Delivery of aa-tRNA to the ribosome

**Figure 1:**
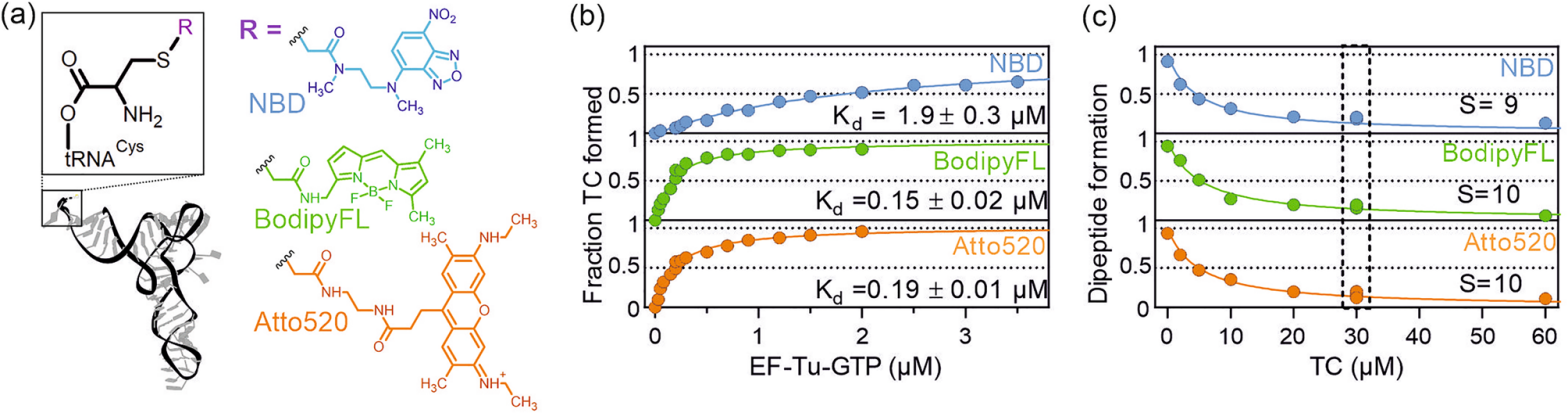
Ternary complex formation with RCys-tRNA and incorporation of RCys into dipeptides. (**a**) Chemical structures of fluorescent probes covalently attached to the amino acid side-chain thiol group to form RCys-tRNA^Cys^. (**b**) TC formation with RCys-tRNA^Cys^ (10 nM) upon addition of increasing concentrations of EF-Tu–GTP monitored by the fluorescence change of the R probe (Figure S2). Data were normalized by setting the fluorescence intensity of RCys-tRNA^Cys^ in the absence of EF-Tu–GTP to 0 and the saturating fluorescence intensity extrapolated by hyperbolic fitting to 1. Lines represent hyperbolic fits using a one site binding model (Material and Methods). The K_d_ value is reported with the standard error of the fit. (**c**) Formation of f[^3^H]Met-RCys dipeptides on 70S IC (20 nM) in the presence of RCys-tRNA^Cys^ (0.12 µM), EF-Tu–GTP (75 µM), and increasing concentration of *E. coli* aa-tRNA without Cys-tRNA^Cys^. f[^3^H]Met-RCys peptides were separated by HPLC (Figure S3). Lines represent fits to a competition model yielding [TC(aa)]_0.5_ as the concentration of TC required to reduce dipeptide formation by half of 4.5 ± 0.6 µM , 5.2 ± 0.4 µM, and 4.5 ± 0.4 µM for NBD, BodipyFL, and Atto520, respectively. The selectivity S for the RCys-tRNA^Cys^ relative to near-cognate aa-tRNAs indicated in the Figure was calculated taking into account the abundance of the near-cognate aa-tRNAs in the total tRNA pool (Methods). For the translation experiments in the next sections [TC(aa)] = 30 µM was kept constant (boxed).

To identify the factors affecting the incorporation of fluorescent nc-aa into proteins, we used an *in-vitro* approach to introduce a fluorescence-labeled Cys into peptides in a fully reconstituted translation system^30^. We labeled the thiol group of enzymatically charged Cys-tRNA^Cys^ with fluorescent probes (R) NBD, BodipyFL or Atto520, which are commonly used dyes for fluorescence labeling of proteins (Fig. 1a). RCys-tRNA^Cys^ was purified by HPLC (Fig. S1) and added to the *in-vitro* translation system lacking native Cys-tRNA^Cys^ (Materials and Methods). This approach allows for testing the cotranslational incorporation of a variety of probes into different nascent sequence contexts, in order to identify steps at which the incorporation fails and potentially circumvent such pitfalls.

One potential limitation to efficient incorporation of nc-aa into peptides is their compromised binding to EF-Tu^22,36,37^. To control for this effect, we monitored the formation of the ternary complex (TC) EF-Tu–GTP–RCys-tRNA^Cys^ using the fluorescence intensity change of the R-group as a readout (Fig. 1b and Fig. S2). Cys-tRNA^Cys^ labeled with BodipyFL or Atto520 exhibits slightly reduced binding affinity to EF-Tu–GTP (0.15 µM and 0.19 µM, respectively) compared to natural aa-tRNA (0.04–0.1 µM)^36,38,39^. The affinity for NBDCys-tRNA^Cys^ is decreased by about tenfold (Fig. 1b). The reduced affinity of EF-Tu to NBDCys-tRNA^Cys^ could potentially cause a bottleneck in NBDCys incorporation into peptides, but this effect can be compensated by the addition of sufficiently high concentration of EF-Tu–GTP into the *in-vitro* translation system.

In the next step, we monitored the incorporation of RCys into f[^3^H]Met-RCys dipeptide in the presence of increasing concentrations of other aa-tRNAs, thereby mimicking the *in-vitro* translation conditions. In the absence of extraneous aa-tRNAs, we observed efficient dipeptide formation for all three RCys-tRNA^Cys^ variants (Fig. 1c). Addition of increasing concentrations of *E. coli* aa-tRNA (without Cys-tRNA^Cys^) decreases the yield of f^3^[H]Met-RCys dipeptides and concomitantly increases the yield of dipeptides formed between f[^3^H]Met and natural aa (Fig. S3). Because the aa-tRNA mixture does not contain cognate Cys-tRNA^Cys^, inhibition of RCys incorporation must be caused by the competition for binding to the Cys codon between the cognate RCys-tRNA^Cys^ and near-cognate (or non-cognate) aa-tRNAs. Based on the rapid equilibrium competition model (Materials and Methods, Model 1), we calculated that the catalytic efficiency k_cat_/K_M_ for the cognate RCys-tRNA^Cys^ is 40 times higher than that for the bulk aa-tRNAs in the mixture^40^. If we consider near-cognate aa-tRNAs as main competitors and assume that the effect of non-cognate aa-tRNAs is negligible, the k_cat_/K_M_ ratio would reduce to a value of only 9–10 because only a limited set of aa-tRNA are near-cognate to Cys-tRNA^Cys^ (see Materials and Methods for further details)^41^. This value is by far smaller than the reported k_cat_/K_M_ selectivity of 30–800 for the native cognate vs. near-cognate aa-tRNA at the initial selection stage^42,43^. Thus, the decoding selectivity for cognate nc-aa-tRNAs appears lower compared to natural aa-tRNA.

### Incorporation of RCys at position 34 of HemK

**Figure 2:**
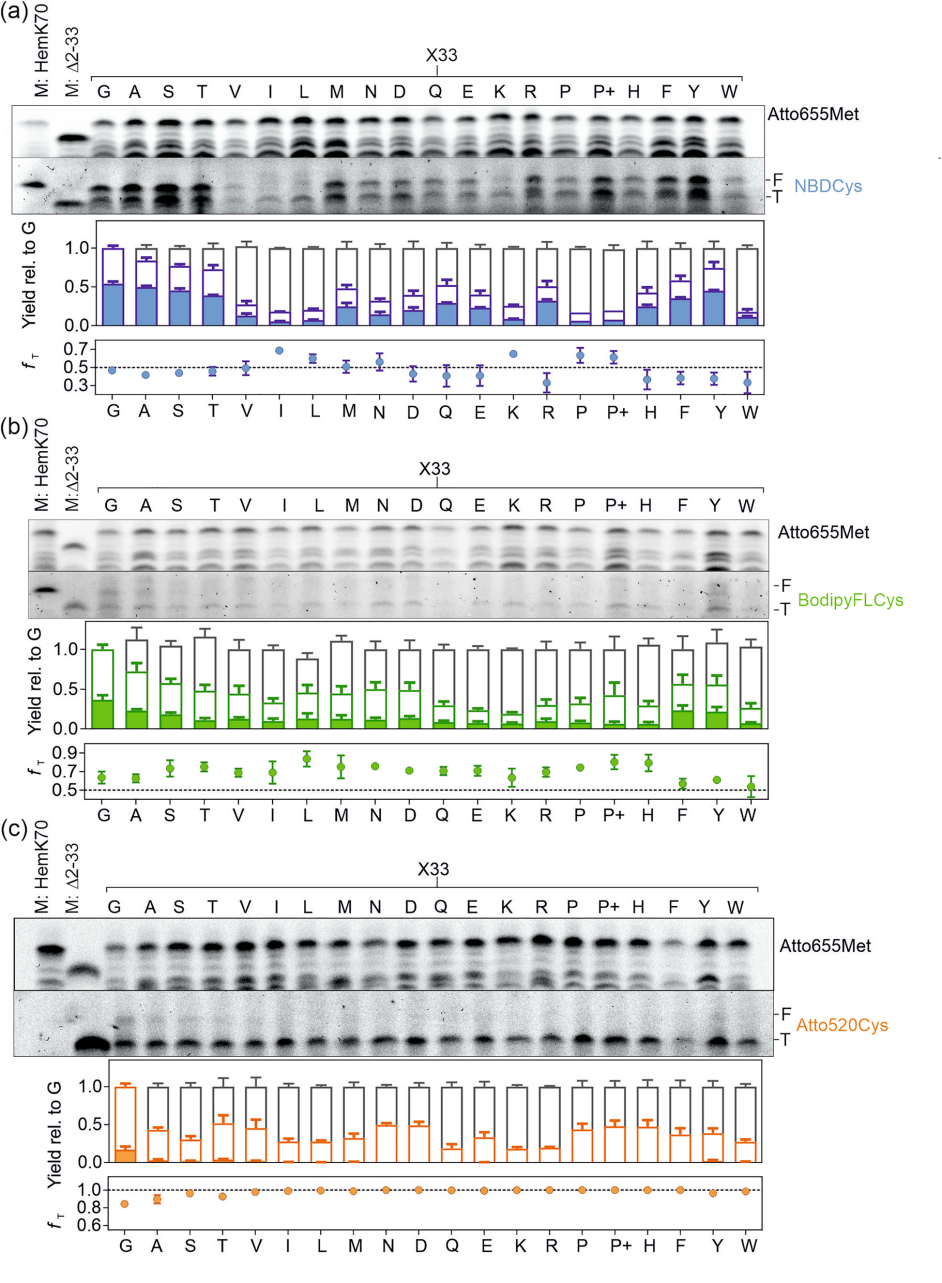
Incorporation of RCys at codon 34 of HemK70: Dependence on the identity of the upstream aa. (**a**) NBDCys incorporation. Upper gel, fluorescence scans of peptide products detecting Atto655-Met1 (top scan) and NBDCys34 (bottom scan) incorporation. Size marker M: HemK70 was prepared by mixing standards prepared individually by initiating HemK70 translation with N-terminal Atto655Met1 or BodipyFLMet1 (Materials and Methods). Size marker M: Δ2-33 was obtained by translating a construct lacking aa 2 to 33 of HemK70 with RCys incorporated immediately after Atto655-Met1 (see also Fig. 4). The incorporation of NBDCys into full length (F) and the truncated (T) HemK70 (bar graph) was quantified relative to the construct with Gly33 using the Atto655 fluorescence in the F product for normalization (Materials and Methods). Low-molecular weight bands in the Atto655 channel represent truncated peptides that appear at limiting concentration of total aa-tRNA. These bands were not considered further, as their mobility is significantly lower than that of the RCys-labeled T product. Stacked bars indicate contributions of T (color-coded open bars) and F (color-coded filled bars); open bars with gray border represent the fraction of peptides that did not incorporate RCys. The fraction of T product (*f*_T_; bottom panel) shows the relative band intensities of T and F products. The construct with Pro33 was translated in the absence (P–) or presence (P +) of EF-P (3 µM). (**b**) and (**c**) same as in (**a**) but for BodipyFL and Atto520, respectively. Error bars indicate the SEM values for n = 5 for NBDCys and BodipyFLCys or n = 4 for Atto520Cys.

In our previous work, we reported efficient incorporation of the large fluorescent probe Bodipy576/589 attached to the ε-side-chain group of Lys-tRNA^Lys^ into position 34 of the model protein HemK containing aa 1–70, HemK70^22^. To have a basis for comparison with the earlier results, we used the same model construct, but replaced Lys at position 34 with Cys to investigate the incorporation of RCys into the protein. The sequence context upstream of the incorporation site can modulate the incorporation efficiency of large nc-aa^18,26^. To systematically asses the influence of the sequence context on the incorporation of fluorescent probes of increasing size, we generated a library of constructs with all possible aa substitutions (except for Cys) at position 33 upstream of the incorporation site (Fig. 2). We used a fully reconstituted *E. coli in-vitro* translation system to synthesize HemK70 in the presence of RCys-tRNA^Cys^ and a large excess of EF-Tu–GTP that is sufficient to overcome the reduced affinity of RCys-tRNA^Cys^ to EF-Tu^22,44^. All experiments were carried out at single-turnover conditions, which ensured that neither the ribosome/mRNA recycling, nor recharging of aa-tRNA played a role in these experiments. In order to allow for normalization of differences in the overall translation efficiency and gel loading among the constructs, we also incorporated the fluorescent probe Atto655 at the N-terminus of HemK70 by performing translation initiation with Atto655-[^3^H]Met-tRNA^fMet^, which does not affect translation^45^. HemK peptides were separated by SDS-PAGE and RCys incorporation was quantified by fluorescence imaging at the excitation/emission wavelength of the specific RCys (Fig. 2).

NBDCys is incorporated into full-length HemK70 with any aa at position 33 (X33; Fig. 2a), but the yield of the full-length (F) peptide carrying NBDCys depends on the identity of the X33 residue. Residues with small side-chains (Gly, Ala, Ser, Thr) and the aromatic residues Phe or Tyr allow for higher yield of NBDCys incorporation into the full-length peptide in comparison to Val, Ile, Leu, or Pro. A sizable fraction of NBDCys is found in the truncated translation product (T) that migrates at similar height as the HemK construct lacking residues 2 to 33 upstream of the incorporation site (M: Δ2-33; Fig. 2a–c), but higher than expected for the N-terminal HemK34 peptide (Fig. S4a). We note that sequence-dependent differences in electrophoretic mobility of such peptide fragments and the addition of fluorescence groups precludes unambiguous assignment of RCys-containing fragments. The fraction of T products is higher and the dependence on the upstream residue is stronger with BodipyFLCys than with NBDCys (Fig. 2b). Incorporation of Atto520Cys into the F product occurred only with Gly33 and to a minor extent with Ala33, whereas in all other cases only T was produced (Fig. 2c). Quantitative assessment using the fraction of truncated translation product relative to total incorporation (*f*_t_ = T/(T + F)) reveals that RCys incorporation into F and T products is modulated differently, e.g., with NBDCys, Ile33 inhibited synthesis of F to a larger extent than T, whereas W33 is less inhibitory for F than for T (Fig. 2a). Addition of EF-P, a factor that accelerates peptide bond formation with Pro residues and enhances the incorporation of D- and β-amino acids^44,46,47^, slightly enhanced the yield of F and T (NBDCys and BodipyFLCys) and of T fragment (Atto520Cys). For all constructs, the incorporation of RCys into F and T products downstream of X33 was lower than into the original Gly33 construct (Fig. 2a–c).

### A steric model based on linear free energy relationships

To gain further insights into the effect of aa at position 33, we used linear free energy relations (LFER)^48^ to correlate the relative incorporation levels for G33X constructs (except for G33P) with aa descriptors for the ground-state properties of the upstream residues. The underlying assumption of this approach is that the yields of products result from the kinetic partitioning governed by the respective rate-limiting steps, which justifies the use of the LFER approach. Mei *et al*.^49^ reported a set of 50 aa descriptors composed of 18 hydrophobic, 17 steric, and 15 descriptors for electronic properties (Table S1). The linear correlations with the aa descriptors were ranked according to the R^2^ value, which indicates how much of the variation in the experimental dataset is captured by the regression model (Fig. S5a–c). In all cases, the steric descriptors exhibited the strongest correlations, whereas the hydrophobic and electronic descriptors did not capture the experimental trends. The steric descriptors are highly correlated among each other (Fig. S4d), which precludes their ranking based on R^2^. However, the steric descriptors can be divided into Taft-type descriptors that parametrize the steric hindrance at the reaction center and those that are related to the overall volume of the side chain^50^. We consistently found a strong correlation with the van-der-Waals volume of the side chain descriptor V35 or the length of the side chain descriptor V23, whereas the correlation with the Taft-type descriptors V21 and V24 for all products was weaker (Fig. S4a–c). For NBDCys incorporation into F product all correlations yielded a lower R^2^ value. This analysis suggests an important role of the overall steric volume of the upstream residue in modulating the incorporation levels of RCys. The incorporation levels calculated based on the coefficients obtained from LFER, e.g., the length of the amino acid side chain, replicate many features of the experimental data (Fig. S4e–g).

The slope of the linear correlation can be interpreted as sensitivity of RCys incorporation towards the given steric descriptor. The absolute values of *b*_*T*_ and *b*_*F*_ depend on the descriptor, e.g. the absolute values for the descriptor V35 representing van-der-Waals volume are larger than V23 for the length of the side chain. Therefore, the slopes obtained for different descriptors cannot be compared to one another. However, each descriptor can be used to compare between T and F formation as well as between different R substituents. For the incorporation of RCys into T products, the slopes *b*_*T*_ for the same descriptor are similar for all RCys, indicating a similar dependence of the rate-limiting step on the properties of the X33 residue. For NBD and BodipyFL the *b*_*F*_* /b*_*T*_ ratio is close to 1, suggesting a similar sensitivity also for F formation. However, for Atto520Cys, the dependence on steric contribution is significantly higher for F than for T (Table S2), which suggests that the rate-limiting steps are different for the two products.

Testing the correlations of log(F) and log(T) with the *E. coli* codon usage frequency and tRNA abundance yielded significantly smaller R^2^ values compared to the steric descriptors (Fig. S5h,i). The yield of the F and T products detected by the RCys fluorescence normalized to the N-terminal Atto655 probe show only a weak dependence on the codon used to encode residue X33. Lastly, in the case of G, R, H, E, F, Y, and K at position 33, the isoacceptor aa-tRNA binds with a similar stability to the P site^51^. Thus, codon-dependent interactions in the P site have only minor, if any, contribution to the yield of F and T products.

### Multiple Gly residues upstream of the incorporation site

**Figure 3:**
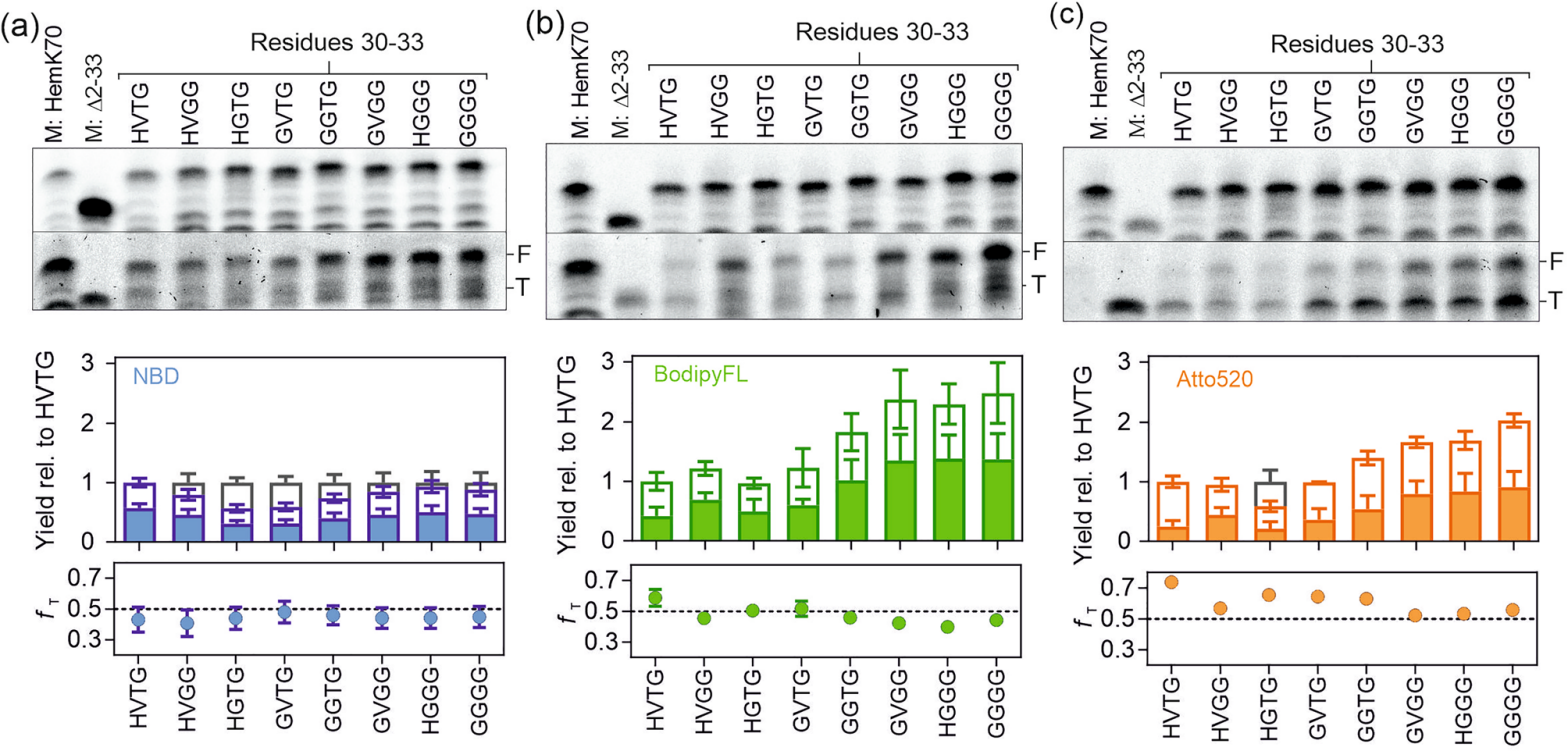
Effect of upstream residues −4 to −2 on RCys incorporation. Separation and quantification of translation products containing Atto655-Met1 and NBDCys34 was performed as described for Fig. 2. M: HemK70 marker was prepared from peptides containing N-terminal BodipyFLMet1 and Atto655Met1. The yield of F (filled bars) and T (open bars) products are shown relative to the native sequence context, HVTG. Error bars indicate the SEM values for n = 5 for NBDCys and n = 6 for BodipyFLCys and Atto520Cys. (a) NBD. (b) BodipyFL. (c) Atto520.

The data in Fig. 2 show that a Gly upstream of the nc-aa incorporation site allowed for the highest RCys incorporation level as well as for the highest fraction of F product among the constructs. An enhanced incorporation of large fluorescent nc-aa was also reported downstream of Gly or Gly-Gly motive in other proteins^19,26^. To systematically investigate whether multiple upstream Gly residues might further enhance the incorporation of RCys, we generated an additional set of mutations in the native HVTG sequence containing Gly substitutions at different positions −1 to −4 relative to the incorporation site. For NBDCys, we did not observe a significant change in the formation of F or T products (Fig. 3a). In contrast, incorporation of BodipyFLCys was much more efficient in the presence of two additional upstream Gly residues in the GVGG, GGTG, or HGGG sequence context. Including a Gly residue at position −4 (GGGG) did not have an additional effect (Fig. 3b). Similarly, constructs with upstream Gly substations increased the incorporation of Atto520Cys, albeit not to the same extent as with BodipyFLCys (Fig. 3c). For both BodipyFLCys and Atto520Cys, the upstream Gly substitutions stimulated formation of both F and T products, resulting in small changes in *f*_*T*_*.* A Gly at position -2 appears to be important, as direct comparison of Gly32 vs. Thr32 constructs consistently showed a higher fraction of the F product for the Gly32 containing sequence. For BodipyFLCys and Atto520Cys, the fraction of the T product slightly decreased with two or more consecutive upstream Gly residues.

### Dependence of RCys incorporation on the upstream sequence length

**Figure 4:**
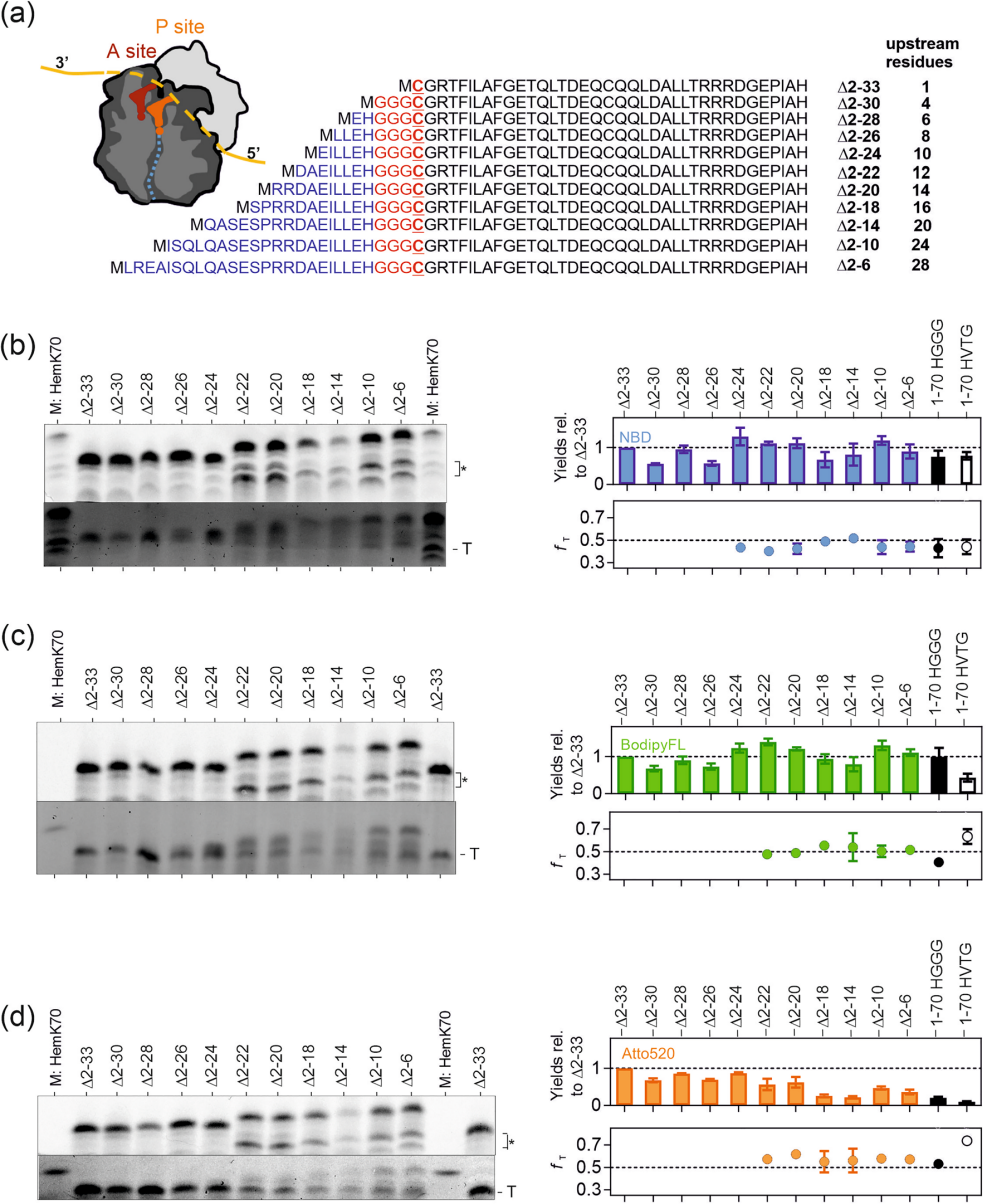
Upstream nascent peptide length effect. (**a**) Sequence of the HemK constructs. All construct except for Δ2-33 contained a GGG sequence upstream of the Cys34 codon. The structural cartoon shows the nascent polypeptide attached to the peptidyl-tRNA in the P site prior to peptide bond formation with RCys-tRNA^Cys^ in the A site. (**b**) Left panel: Visualization of nascent chains containing Atto655Met1 (top gel) or NBDCys (bottom gel) by SDS-PAGE as described in Fig. 2. An asterisk indicates short peptide products resulting from ribosome stalling prior to codon 34. Right panel: The sum of F and T products (NBDCys signal) for different upstream nascent chain lengths, shown relative to that for the shortest construct Δ2-33 (top panel) and the *f*_T_ values (bottom panel). The values for HemK 1–70 HGGG (from Fig. 3) and HemK 1–70 HVTG (from Fig. 2) are shown as black or white symbols, respectively. (**c**) and (**d**) are same as (**b**) for BodipyFLCys and Atto-520-Cys, respectively, with the exception that marker M:HemK70 contained only peptides with the N-terminal BodipyFL.

Lastly, we tested how the length of the nascent peptide upstream of the labeling site affects RCys incorporation. We generated a set of constructs with amino acid deletions upstream of the HGGG sequence preceding the RCys34 incorporation site (Fig. 4a). Notably, the critical −4 to −1 residues remain at the same location in the exit tunnel at the time when RCys is incorporated and the differences between the nascent chain complexes are only in the distal N-terminal part of the nascent chain.

A single major peptide product carrying the N-terminal Atto655-Met1 appears when the nascent chain upstream of Cys34 is up to 10 aa in length (Δ2–24) (Fig. 4b–d, top gel scans). The gel scans for RCys labels also show a corresponding peptide. Although the electrophoretic mobility of the product hardly changes with size for the increase in upstream sequence length from 1 to 10 aa (total peptide size of 35 to 45 aa), we note that a translation stop immediately after RCys incorporation would result in very short peptides (2 to 11 aa) that would be probably too small to detect on gels. Thus, RCys incorporation most likely does not inhibit translation of the mRNA sequence downstream of codon 34. As the sequence upstream of Cys34 expands from 12 to 28 aa, the mobility of the upper peptide band changes in both the Atto655-Met1 and RCys34 channels, as expected for a chain length increasing from 47 aa in the Δ2-22 construct to 65 aa for Δ2-6 in total (Fig. 4b–d). With these constructs, we observe pronounced ribosome pausing manifested by appearance of discrete bands marked by a bracket in Fig. 4b–d below the respective full-length Atto655-Met1 band. The mobility of the peptides arising from pausing also depends on the sequence length upstream of HGGC, suggesting that these peptides represent N-terminal peptide fragments. Although it is not clear why these fragments arise, they are notably shorter than the control Δ2-33 peptide (38 aa) and are most likely drop-off products of translation prior to codon 34. If translation stalled after the incorporation of RCys into the nascent peptide, the respective peptides (2 to 34 aa length) should be also found in this region, but such bands are not detectable in the RCys channel (lower panels). Instead, RCys is present in a band that migrates at a similar height as the Δ2–33 peptide independent of the overall construct length as well as in high molecular weight bands that migrate dependent on the construct length. The RCys-containing band that is independent of construct length does not give rise to a band in the Atto655-Met1 channel and thus most likely represents the C-terminal peptide fragment that starts from RCys, whereas the top band entails the double-labeled peptides of respective length together with those labeled with Atto655-Met1 alone. The *f*_T_ value for the Δ2–22 to Δ2–6 constructs depends on the dye, e.g. it is 0.45–0.51 for NBD, 0.41–0.55 for BodipyFL and 0.53–0.61 for Atto520, but is essentially independent of the construct length (Fig. 4b–d, right panels).

To compare the relative incorporation levels of RCys into constructs of different length, RCys fluorescence was normalized to that of the shortest construct Δ2-33 where RCys is incorporated as second residue adjacent to Atto655-Met1, which allows for the comparison to the absolute incorporation levels of RCys established in Fig. 1c (see Methods). For NBDCys and BodipyFLCys, the total incorporation into the full-length and truncated translation products did not change considerably (i.e., by more than a factor of 2) upon truncation of the N-terminal sequence in the HGGG context (Fig. 4b,c, right panels; the only exception is Δ2-14 where the intensity for Atto655-Met1 and all RCys is reduced for unknown reasons). In contrast, the incorporation of Atto520Cys in F and T gradually decreased with increasing length of the peptide sequence upstream of the HGGG sequence (Fig. 4d, right panel). With longer upstream polypeptide sequences, the HGGG motif supported very little Atto520Cys incorporation, whereas the *f*_*T*_ ratio remained constant, suggesting that the presence of a longer nascent chain did not affect the partitioning into F and T products but rather decreased the overall incorporation of Atto520Cys, potentially by favoring incorporation of the natural aa.

## Discussion

**Figure 5:**
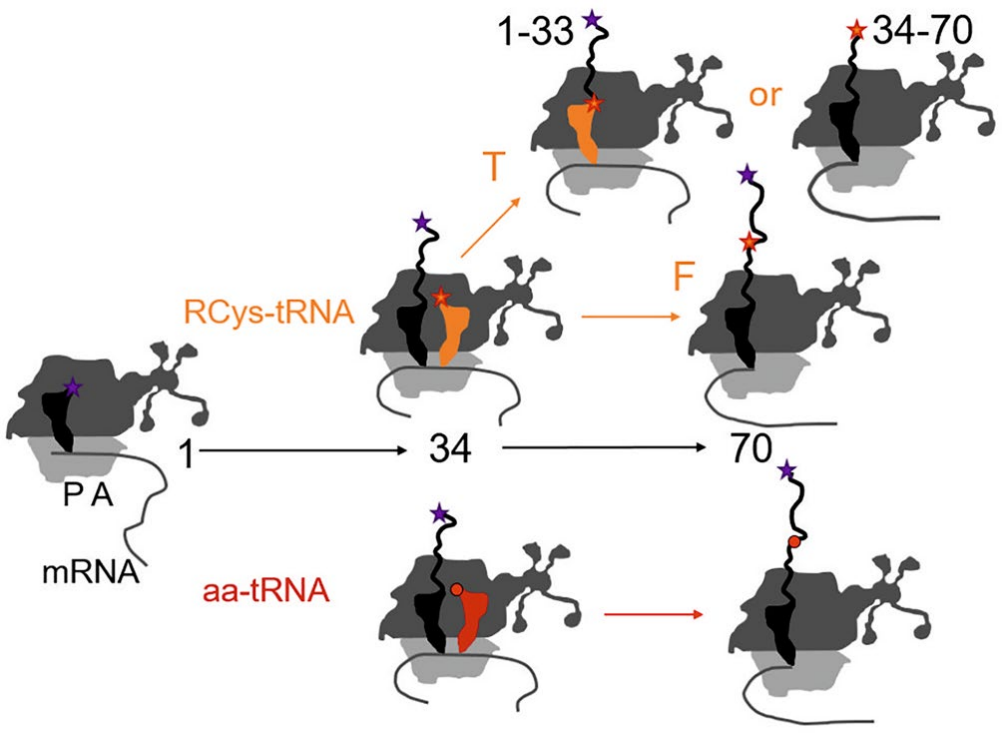
RCys incorporation scenarios. Synthesis of HemK70 begins with 70S initiation complex, in the present experiments containing Atto655-Met1-tRNA^fMet^ in the P site. At position 34, RCys-tRNA^Cys^ (orange) or a competing aa-tRNA (red) is incorporated and translation elongation can proceed to full-length HemK70 or result in formation of truncated products. If the ribosome stalls after peptide bond formation, the resulting T product contains RCys in the N-terminal fragment. Alternatively, stalling might occur before peptide bond formation between X33 and RCys34; the following translocation of RCys-tRNA^Cys^ to the P site then results in labeling of the C-terminal fragment, akin to the products of the StopGo translation in eukaryotes.

Our data on RCys incorporation are consistent with a simple model that predicts three likely outcomes of translation at codon Cys34 (Fig. 5). When codon 34 arrives at the A site of the ribosome, the P site is occupied with the peptidyl-tRNA, e.g. peptidyl-Gly-tRNA^Gly^ in case of the native HemK mRNA sequence. The ribosome then accepts either the cognate RCys-tRNA^Cys^ or a near-cognate native aa-tRNA owing to the low selectivity of the ribosome for RCys-tRNA^Cys^. Misreading of an internal codon by a near-cognate aa-tRNA in most cases does not halt translation; as a results, full-length peptides are produced. Our estimations of the ribosome selectivity for the RCys- vs. other aa-tRNAs suggest that depending on the relative concentrations of labeled and unlabeled aa-tRNAs, a large fraction of ribosomes may favor a near-cognate aa-tRNA, thereby reducing the yield of fluorescence-labeled product (Fig. 1c). Notably, the selectivity for the Bodipy576/589-labeled Lys-tRNA^Lys^ appears to be somewhat higher, as we were able to achieve 70% incorporation efficiency of Bodipy576/589 at codon 34 of HemK^52^. Using genetically engineered tRNAs, e.g., with a tetranucleotide anticodon, would likely increase the selectivity for the respective cognate nc-aa-tRNA, as there are no natural cognate or near-cognate tRNAs for the respective four-letter codon. However, in this case an occasional misreading of a three-letters codon by a native aa-tRNA may result in ribosome frameshifting, thereby abolishing synthesis of the desired polypeptide^53^.

Successful accommodation of RCys-tRNA in the A site completes the decoding phase, but does not necessarily result in continuing translation (Fig. 5). Only a fraction of ribosomes proceeds to synthesize the full-length protein, as indicated by the *f*_*T*_ fraction of the truncated relative to the full-length peptides. In principle, the observed truncated products may arise if ribosomes stop elongation immediately after incorporation of RCys^54–56^; in this case, the N-terminal 1–34 aa HemK fragment would be RCys-labeled at its C-terminus. However, we find truncated peptides that do not carry the N-terminal label (Fig. 4), indicating that they lack the N-terminal part. One possible scenario is that a defect in peptide bond formation may arrest ribosomes in a state with a peptidyl-tRNA in the P site and RCys-tRNA^Cys^ in the A site. At this point in translation, the peptide moiety must be released from the P-site tRNA to allow translocation of RCys-tRNA^Cys^, because a peptidyl-tRNA with a long nascent chain is unlikely to translocate due to interactions of the nascent peptide with the exit tunnel (see also below). The subsequent tRNA translocation would then release deacylated tRNA, whereas ribosomes would continue translation of the HemK mRNA producing the 34–70 aa peptide that is labeled at the N-terminus with RCys (Fig. 5). Similar observations have been reported for the incorporation of other large fluorescent probes, D-aa, and aromatic oligoamines^18,57,58^.

Large nc-aa were reported to be more efficiently incorporated downstream of aa residues with small side chains such as Gly residues^19,26^. In agreement with this notion, we find that the aa context upstream of Cys34 has a strong effect on the yield of RCys incorporation and Gly is the best penultimate residue; other aa can be tolerated depending on the nature of R. With NBD, a number of other aa are tolerated at the upstream position, whereas with BodipyFL and particularly Atto520, Gly is the only residue that allows for significant RCys incorporation. This effect is unlikely to be due to the competition between RCys-tRNA^Cys^ and near-cognate aa-tRNAs at the initial selection phase of decoding, which is dominated by codon recognition on the small ribosomal subunit. In contrast, the accommodation of RCys-tRNA^Cys^ in the A site of the large ribosomal subunit may depend on the nature of the side chain occupying the P site, e.g. a bulky side chain could provide a steric hindrance to positioning of RCys in the A site and favor rejection of RCys-tRNA prior to peptide bond formation. In fact, our LFER analysis (Fig. S5) indicates that the efficiency of RCys incorporation depends on steric properties of the X33 side chain such as the side chain length and van-der-Waals radius. Furthermore, after RCys-tRNA accommodation only a fraction of it appears in full-length peptides, indicating additional defects at the step of peptidyl transfer (Fig. 5). This notion is particularly true for Atto520Cys, which shows a higher sensitivity to the steric properties of X33 residues for synthesis of F protein compared to the T fragment (Table S2).

In addition to Gly33 at the -1 position, Gly residues at positions −2 to −4 further enhance the incorporation of BodipyFLCys and Atto520Cys (Fig. 3). Stretches of consecutive Gly residues may make the nascent peptide more flexible and leave more space inside the ribosomal exit tunnel to accommodate bulky nc-aa and facilitate better positioning of RCys for catalysis. With BodipyFLCys and Atto520Cys, the simultaneous increase in RCys incorporation into both F and T (Fig. 2) might indicate that the upstream Gly residues inside the ribosome exit tunnel modulate the RCys-tRNA accommodation in the A site (Fig. 5). In addition, the decrease in the *f*_*T*_ value indicates that synthesis of the full-length protein is enhanced, suggesting that multiple Gly residues have a favorable effect on peptide bond formation. Thus, protein engineering to accommodate Gly-rich stretches upstream of the incorporation site might be a generic solution to increase the incorporation of large nc-aa by decreasing the steric hindrance in the peptidyl transferase center. Of note, by analogy to the enhancement of large nc-aa incorporation, a Gly residue in the P site also allows for flexible orientation of antibiotics chloramphenicol and oxazolidinone in the A site of the peptidyl transferase center in a manner that overcomes the inhibition by promoting drug displacement by the accommodating A-site tRNA^59^.

Our data also show that RCys incorporation strongly depends on the length of the nascent peptide upstream of the incorporation site. Large nc-aa can be more readily incorporated at N-terminal positions of the nascent chain than at the middle position in the protein^24^. Even for the largest probe Atto520, we observe an efficient labeling at the second position of the nascent chain, which decreases dramatically with the chain length (Fig. 4). Interactions of the nascent chain with the exit tunnel may induce a conformation of the peptidyl transferase center that disfavors nc-aa-tRNA accommodation^60^ or reduce the mobility of the peptidyl-tRNA thereby preventing sampling of the conformation that allow peptide bond formation^61,62^. In contrast, for canonical aa the presence of a longer nascent chain attached to the peptidyl-tRNA can improve the positioning of the P-site substrate. This is manifested by the acceleration of peptide bond formation with nascent peptide length as well as restoration of translation elongation for P-site tRNA lacking the 2’OH group on the terminal adenine A76^63,64^.

The formation of T products that entail the C-terminal part of the protein and start from the fluorescence labeled RCys (Fig. 4) is reminiscent of the StopGo translation (also known as ribosome “skipping” or “stop-carry on”) in eukaryotic cells mediated by the viral 2A sequence^65,66^. Specific elements of the 2A nascent peptide promote ribosome stalling before incorporation of Pro-tRNA^Pro^ into the nascent peptide. Presumably, the release of the nascent peptide from the P-site tRNA is promoted by translation termination factors 1 and 3. Then, Pro-tRNA^Pro^ is translocated from the A site to the P site and the ribosome continues protein synthesis. StopGo translation has not been described in prokaryotes, but StopGo-like translation events were observed in bacterial cell-free translation systems upon incorporation of nc-aa with bulky side chain structures, such as large fluorescent probes or bulky aromatic oligo amides^18,58,67^, D-Trp and D-Arg^57^ and Lβ-homoglycine-Trp^68^. It is unclear what happens to the P-site peptidyl-tRNA in the bacterial system, as termination factors are not present in our reconstituted translation assay and thus the mechanism must differ from that of the authentic StopGo. In principle, peptidyl-tRNA could dissociate from the ribosome, but the rate of peptidyl-tRNA drop-off decreases dramatically when peptides grow more than 4 to 6 aa in length^69,70^. Importantly, a similar level of T product was formed upon shortening the upstream nascent chain length from 28 to 12 residues (Fig. 4), indicating that a shorter peptide—which is presumably more prone to drop-off—does not stimulate the StopGo-like event. Similarly, a high level of a StopGo-like translation product was reported upon incorporation of TAMRA fluorescent probe for a nascent chain with 22 aa upstream of the StopGo site^18^. These findings argue against a peptidyl-tRNA drop-off event during the formation of truncated translation products while incorporating large nc-aa. Alternatively, peptidyl-tRNA might undergo hydrolysis on the ribosome forming a pretranslocation complex with a deacylated tRNA in the P site and aa-tRNA in the A site. Such ribosomes can perform translocation, albeit slowly^71,72^. Spontaneous peptidyl-tRNA hydrolysis is typically much slower than the steps of the on-going translation elongation^73^. However, a long pause due to delayed accommodation of RCys-tRNA^Cys^ may open the access of water to the catalytic site and promote hydrolysis. Thus, incorporation of large non-canonical fluorescence probes may be inefficient not only because they compete with highly abundant natural aa-tRNAs, but also due to inherent defects in accommodation and peptide bond formation that can reprogram the peptidyl transferase center from peptide bond formation to non-canonical termination activity. Screening the 23S rRNA libraries focusing on residues 2057–2063 and 2492–2507 at the peptidyl transferase center yielded ribosome variants that enhanced the incorporation of β-aa, dipeptides, dipeptidomimetic, and cyclic amino acids^74,75^. As some dipeptidomemetics approach the molecular weight of NBDCys and BodipyFLCys, it would be interesting to test whether such mutant ribosomes avoid premature termination. Our results also suggest that nc-aa with large or bulky side chains have a potential to act as 2A sequence analogs for coexpression of multiple proteins in bacteria from a single open reading frame, thereby pointing at an unexpected biotechnological potential of nc-aa.

## Materials and methods

### Buffers and reagents

All chemicals were purchased from Merck and Sigma Aldrich. Radioactive amino acids were from Hartmann Analytic. Iodoacetamide-NBD amide (NBD) and BodipyFL-C_1_-iodoacetamide (BodipyFL) were purchased from Thermofisher Scientific. Atto520-iodoacetamide and Atto655-NHS were obtained from Atto-Tec. Buffer A: 50 mM Tris–HCl, pH 7.5, 70 mM NH_4_Cl, 30 mM KCl, 7 mM MgCl_2_. Buffer B: 50 mM Tris–HCl, pH 7.5, 70 mM NH_4_Cl, 30 mM KCl, 3.5 mM MgCl_2_, 0.5 mM spermidine, 8 mM putrescine, 2 mM DTT. Buffer C: 100 mM HEPES pH 7.5, 15 mM KCl, 7 mM MgCl_2_. Buffer D: 20 mM NH_4_OAc-HOAc pH 4.5, 10 mM MgCl_2_, 400 mM NaCl, 5% EtOH (v/v). Buffer E: 20 mM NH_4_OAc pH 4.5, 10 mM MgCl_2_, 400 mM NaCl, 40% EtOH (v/v).

### Ribosomes, translation factors and total aa-tRNA without Cys-tRNA^Cys^

Ribosomes from *E. coli* MRE 600, initiation factors (IF1, IF2, IF3), and elongation factors (EF-G, EF-P, EF-Tu) were prepared as described^44,76–78^. Total tRNA from *E. coli* MRE 600 (Roche) was aminoacylated with 19 amino acids omitting L-Cys^79^.

### Preparation of RCys-tRNA^Cys^

*E. coli* tRNA^Cys^_GCA_ was subcloned into pUC19, transcribed in vitro*,* and purified^30^. Aminoacylation was carried out in buffer C with DTT (5 mM), ATP (2 mM), L-Cys (150 μM), tRNA^Cys^ (35 A_260_ units mL^−1^), and S-100 enzymes (6%) for 45 min at 37 °C. Labeling of Cys-tRNA^Cys^ (30 µM) was performed with different fluorescent probes (1 mM) in 50 mM HEPES pH 7.5, 50% DMF for 45 min at 25 °C^30^. Unreacted BodipyFL and Atto520 was removed by extraction with 50:50 phenol:chloroform followed by ethanol precipitation. Excess of the more hydrophilic NBD was removed by size-exclusion chromatography using a G-25 NAP-5 column (GE Healthcare). Labeled aa-tRNA was separated by HPLC using a LiChrospher WP300 RP-18 column and a step-gradient from buffer D to buffer E at a flow rate of 0.5 ml min^−1^: 15 min, 0% buffer E; 2 min, 0–30% buffer E; 20 min, 30% buffer E constant; 30 min, 30–100% buffer E. The concentration of RCys-tRNA^Cys^ was determined by the absorbance of the R group.

### Preparation of Atto655- [3H]Met-tRNAfMet

[^3^H]Met-tRNA^fMet^ was prepared as described^76^. To obtain Atto655-[^3^H]Met-tRNA^fMet^, [^3^H]Met-tRNA^fMet^ (50 µM) was reacted with Atto655-NHS (2 mM) in 100 mM NaOAc pH 5.0, 80% DMSO for 18 h at 4 °C. Unreacted fluorophore was removed by extraction with 50:50 phenol:chloroform followed by ethanol precipitation. Atto655-[^3^H]Met-tRNA^fMet^ was purified by HPLC as described for RCys-tRNA^Cys^.

### mRNA Constructs

HemK cloned into pET24a (Novagen) was used as template for site-directed mutagenesis^52^ using oligonucleotide primers listed in Table S3. In order to minimize bias by codon usage and wobble decoding, the codon cognate to the most abundant isoaccepting tRNA pairing with Watson–Crick geometry was selected for substitution at position 33 of HemK^41^. For constructs with additional Gly substitutions codons GGU and GGC were used that are decoded by the major isoacceptor tRNA^Gly3^. mRNA used for RCys incorporation of RCys or translation of size-marker were prepared as described^44^.

### Determination of RCys-tRNA^Cys^ Affinity to EF-Tu–GTP

In order to form the binary complex EF-Tu–GTP, EF-Tu–GDP (40 µM) was incubated with GTP (2 mM), phosphoenol pyruvate (PEP) (6 mM), and pyruvate kinase (0.1 mg ml^−1^) in buffer B at 37 °C for 15 min. MgCl_2_ was added to compensate for GTP- and PEP-complexed Mg^2+^^80^. EF-Tu–GTP was added to RCys-tRNA^Cys^ (10 nM) and incubated for 2 min at 37 °C and the fluorescence change monitored in the Fluorolog spectrofluorimeter (Horiba Jobin Yvon). NBD emission was monitored at 525 nm after excitation at 480 nm. BodipyFL emission was monitored at 510 nm after excitation at 470 nm. Atto520 emission was monitored at 545 nm after excitation at 500 nm. The fluorescence change $\mathrm{\Delta F}$ was fitted to a hyperbolic binding model:

$$\mathrm{\Delta F }={\mathrm{F}}_{0}+{\mathrm{\Delta F}}_{max}\frac{[EF-Tu]}{{K}_{d}+\left[EF-Tu\right]},$$

where *F*_*0*_ as initial fluorescence, ${\mathrm{\Delta F}}_{max}$ the maximum fluorescence change and ${K}_{d}$ is the equilibrium dissociation constant. The respective values are reported in Fig. 1b.

### 70S initiation complex formation

Initiation complexes were formed by incubating 70S ribosomes (1.5 µM) with initiation factors IF1, IF2, IF3 (1.5 µM each), mRNA (3 µM), GTP (1 mM) and either f [^3^H]Met-tRNA^fMet^ (1 µM) or Atto655-[^3^H]Met-tRNA^fMet^ in buffer A for 60 min. Initiation complex formation was monitored by nitrocellulose filtration and radioactivity counting.

### Dipeptide formation

Ternary complexes were formed in buffer B by incubating EF-Tu–GTP (75 µM) with RCys-tRNA^Cys^ (120 nM) (TC(RCys)), with or without total aa-tRNA lacking Cys-tRNA^Cys^ (0–60 µM) (TC(aa)) for 2 min at 37 °C. Dipeptide formation was initiated by the addition of TC to IC (20 nM) containing 70S ribosomes, f[^3^H]Met-tRNA^fMet^ and mRNA HemK70 Δ2-33 K34C. The reaction was carried out for 15 min at 37 °C. The antibiotic viomycin (200 µM) was added to the dipeptide reactions in order to stop translocation after a single round of peptide bond formation. The samples were flash-frozen, then thawed in the presence of 0.4 mg mL^−1^ RNaseA (Thermo Scientific) and digested for 30 min at 37 °C. The pH value was adjusted by the addition of 1/10 volume of ice acetic acid. Dipeptides were separated by HPLC using a LiChrospher 100 RP-8 column and a gradient from 0 to 65% ACN in 0.1% TFA: 5 min, 0% ACN; 30 min, 0 to 65% ACN; 5 min, 65% ACN. The selectivity (S) of TC(RCys) for binding to the Cys codon relatively to the near-cognate aa-tRNAs were estimated based on the rapid equilibrium competition model:
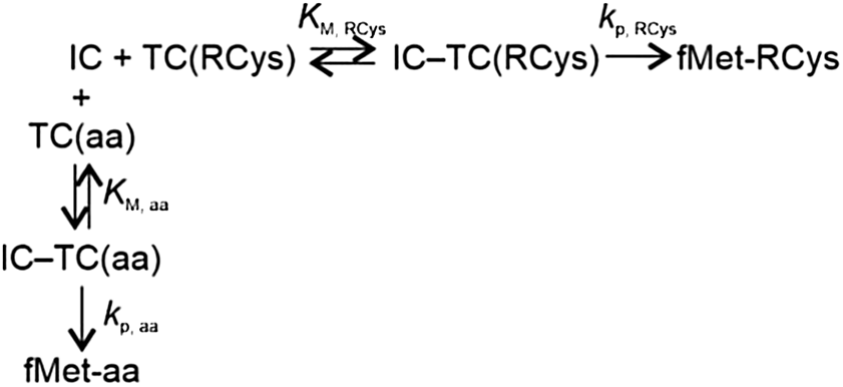


**Model 1. Rapid equilibrium competition model for incorporation of RCys and natural aa into dipeptides.** *K*_*M*_ denotes the Michaelis–Menten constant and *k*_*p*_ the rate of peptide bond formation. The fraction *f*_RCys_ represents the fraction of ribosomes that incorporated RCys into the dipeptide. [TC(aa)]_0.5_ corresponds to the concentration of total TCs required to reduce f_RCys_ to 0.5 as determined in Fig. 1c.

For a mixture of TC(RCys) and TC(aa), the fraction of IC that formed the dipeptide ${f}_{RCys}$ is determined by the Eq. 1:

1 $${f}_{RCys}=\frac{{k}_{p, RCys}\frac{[TC\left(RCys\right)]}{{K}_{M, RCys}}}{{k}_{p, RCys}\frac{[TC\left(RCys\right)]}{{K}_{M, RCys}}+ {k}_{p, aa}\frac{[TC\left(aa\right)]}{{K}_{M, aa}}},$$

where ${k}_{p, RCys}\; \mathrm{ and}\; {k}_{p, aa}$ are incorporation rates of RCys and aa, respectively, into the dipeptide and ${K}_{M, RCys} \mathrm{ and}$ ${K}_{M, aa}$ are Michaelis–Menten constants for the TC(RCys) and TC(aa), respectively. The terms in square brackets are the respective concentrations. TC(aa)_near-cognate_ was calculated from the total aa-tRNA TC(aa) concentration assuming that aa-tRNAs near-cognate to the Cys codon (tRNA^Arg2^﻿, tRNA^Gly3^_,_ tRNA^Phe^_,_ tRNA^Ser3^_,_ tRNA^Sec^_,_ tRNA^Ser5^_,_ tRNA^Trp^_,_ tRNA^Tyr1^, and tRNA^Tyr2^) constitute about 24% of total *E. coli* tRNA^41^. From, Eq. 1, at the concentration of total TC required to reduce ${f}_{RCys}$ by half ([TC(aa)]_0.5_),

2 $${k}_{p, RCys}\frac{[TC\left(RCys\right)]}{{K}_{M, RCys}}={k}_{p, aa}\frac{{[TC\left(aa\right)]}_{0.5}}{{K}_{M, aa}},$$

and the selectivity S for the RCys incorporation, which is proportional to $\frac{{k}_{p, RCys}}{{K}_{M, RCys}}\frac{{K}_{M, aa}}{{k}_{p, aa}}$ equals to

3 $$S= \frac{{[TC\left(aa\right)]}_{0.5}}{[TC\left(RCys\right)]}.$$

The respective S values calculated from [TC(aa) _near-cognate_]_0.5_ are reported in Fig. 1c.

### In vitro translation and analysis

Translation in the fully reconstituted single-turnover in vitro translation system was carried out as described^22,44^. 70S IC (20 nM) containing Atto655-[^3^H]Met-tRNA^fMet^ was mixed with EF-G (3 µM) and TCs formed from EF-Tu–GTP (75 µM), total aa-tRNA without Cys-tRNA^Cys^ (30 µM), and RCys-tRNA^Cys^ (120 nM) in buffer B and incubated for 15 min at 37 °C. To study the incorporation of BodipyFLCys or Atto520Cys, reactions were quenched by the addition of Laemmli sample buffer. Peptidyl-tRNA and aa-tRNA were subjected to aminolysis by 1.5 M NH_2_OH for 16 h at 37 °C^81^. To study NBDCys incorporation, the reactions were stopped by flash freezing, thawed in the presence of 0.4 mg mL^−1^ RNaseA and digested for 30 min at 37 °C. Nascent polypeptides were resolved by Tris-tricine SDS-PAGE using 4% stacking, 10% spacer and 16.5% separation gels (49.5% T, 3% C) and scanned on a FLA-9000 fluorescence imager (Fujifilm Lifescience). Fluorescence of NBDCys and BodipyFLCys was excited at 473 nm and monitored after passing a 510 nm long pass filter. Fluorescence of Atto520Cys was excited at 532 nm and monitored after passing a 575 nm long pass filter. Fluorescence of Atto655 was excited at 685 nm and monitored after passing a 700 nm band pass filter. All scans were saved as 16 bit grayscale image. For the full-sized gel images in Fig. S6–S9, a constant linear contrast 270 to 6868 was applied to all 16 bit images. Size markers (highlighted as M) was generated by *in-vitro* translation of HemK70 mRNA (or Δ2-33 construct) using BodipyFL-[^3^H]Met-tRNA^fMet^ or Atto655-[^3^H]Met-tRNA^fMet^ as initiator tRNA^44^. The two fluorescence-labeled markers were produced individually, mixed, and used as SDS-PAGE markers that gave signals in both Atto655 and NDB/BodipyFL channels. Band intensities of all products was quantified using ImageJ (Version 1.51 23)^82^. Background subtraction was performed globally for all samples on the same gel. To account for translation and gel loading variations, the intensity of F and T bands was corrected by the band intensity of full-length HemK70 in the Atto655 channel and all resulting values further normalized to the RCys signal of the G33 construct. The relative fraction of the full-length product that does not contain RCys is calculated as 1 − (F + T) after the normalization. The fraction of truncated product *f*_T_ was calculated as T/(F + T). Band intensities of peptides with N-terminal truncations (Fig. 4) were normalized to the intensity of the Δ2-33 construct.

### Linear regression for correlation with amino acid descriptors

Linear regression was performed using the equation $y=a+b\times V$ with *y* as log_10_ of the RCys incorporation into F or T, *a* as off-set, *b* as coefficient (slope), and *V* as aa descriptors from Table S1 that can also be retrieved in part from the AA index database^83^. Abundance of *E. coli* tRNA was from ref ^41^ and codon usage frequencies from the Kazusa codon usage database (https://www.kazusa.or.jp/codon/). Linear regressions and correlations between aa descriptors not contained in the AA index database were analyzed with the R software package (version 3.5.3).

## Supplementary Information

Supplementary Information.
