# Supplementary material for: Ribosome selectivity and nascent chain context in modulating the incorporation of fluorescent non-canonical amino acid into proteins
Source: Sci Rep. 2022 Jul 27;12:12848. doi: 10.1038/s41598-022-16932-7 (PMC9329280; doi:10.1038/s41598-022-16932-7)
Supplement: Supplementary file 1 — Supplementary Information. [file 41598_2022_16932_MOESM1_ESM.pdf]

Supplementary Information for

Ribosome selectivity and nascent chain context in modulating the incorporation of fluorescent non-canonical amino acid into proteins

Michael Thommen[1‡], Albena Draycheva[1‡], and Marina V. Rodnina[1*]

[1]Department of Physical Biochemistry, Max Planck Institute for Multidisciplinary Sciences, Göttingen, Germany

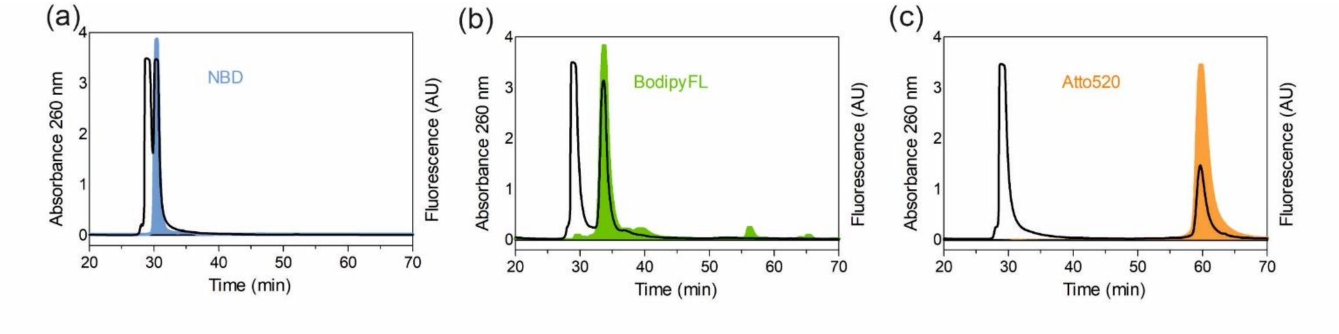

Figure S1. Purification of RCys-tRNA^{Cys} by reversed-phase HPLC.

(a) Separation of NBDCys-tRNA^{Cys} from unlabeled Cys-tRNA^{Cys} and deacylated tRNA^{Cys} was monitored by absorption at 260 nm (black line) and the fluorescence emission of the NBD probe at 530 nm after excitation at 476 nm (blue area).

(b) Same as a) for BodipyFLCys-tRNA^{Cys}. BodipyFL emission was monitored at 580 nm after excitation at 500 nm (green area).

(c) Same as a) for Atto520-Cys-tRNA^{Cys}. Atto520 emission was monitored at 600 nm after excitation at 516 nm (orange area).

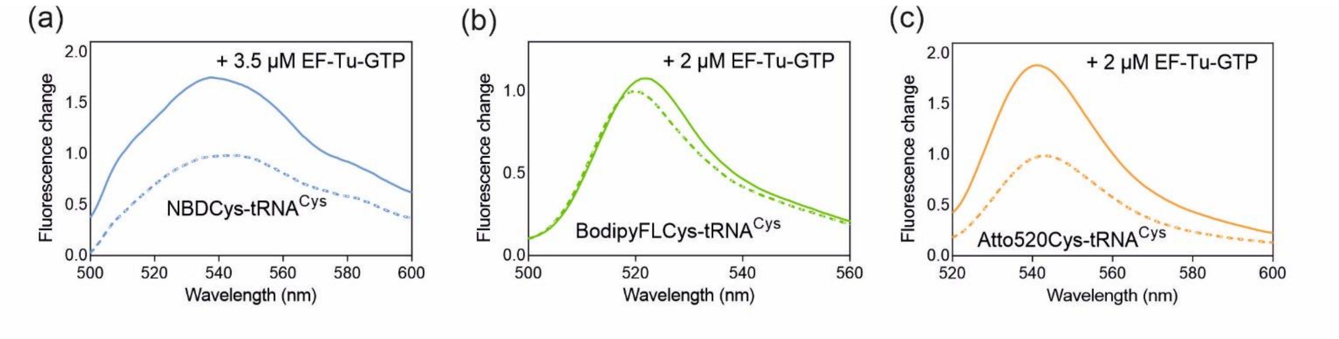

Figure S2. Fluorescence emission spectra of RCys-tRNA^{Cys} in the absence and presence of EF-Tu–GTP. (a) NBD-Cys-tRNA^{Cys}. (b) BodipyFLCys-tRNA^{Cys}. (c) Atto520Cys-tRNA$^{Cys.}$ The concentration of EF-Tu–GTP corresponds to the final concentration in Figure 1b.

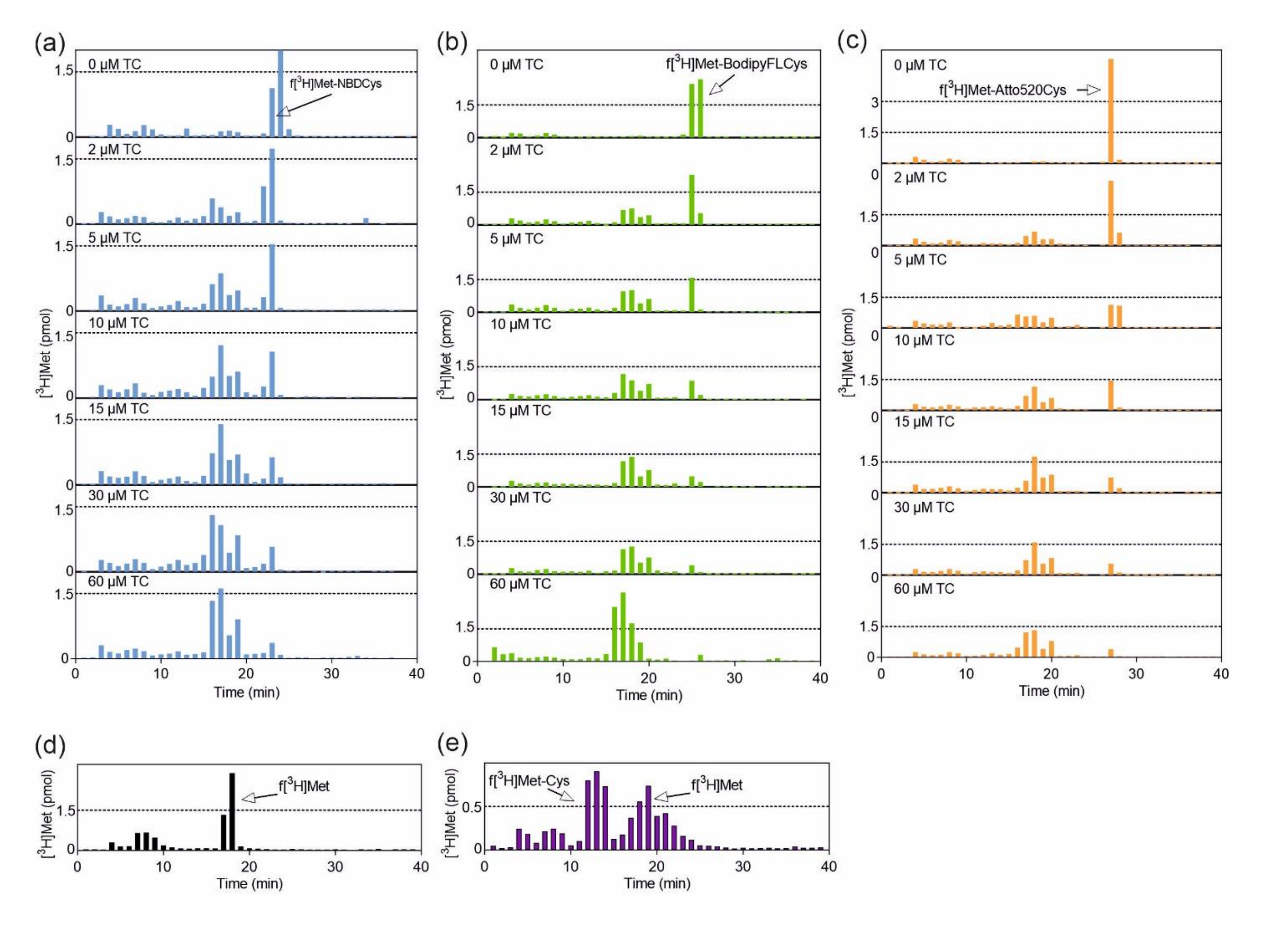

Figure S3. HPLC separation of f [^{3}H]]Met-RCys and f [^{3}H]]Met-aa dipeptides. The identity of the near-cognate aa incorporated into the dipeptide is unknown.

(a) Chromatographic separation of dipeptides formed in the presence of NBDCys-tRNA^{Cys} and increasing concentrations of EF-Tu–GTP–aa-tRNA. The retention time of the f[^{3}H]Met-RCys dipeptides are indicated.

(b) same as (a) for BodipyFLCys-tRNA^{Cys}

(c) same as (a) for Atto520-Cys-tRNA$^{Cys.}$.

(d) Control reaction in the absence of RCys-tRNA^{Cys} or TC.

(e) Dipeptide formation with unlabeled EF-Tu–GTP–Cys-tRNA^{Cys} in the absence of other TCs.

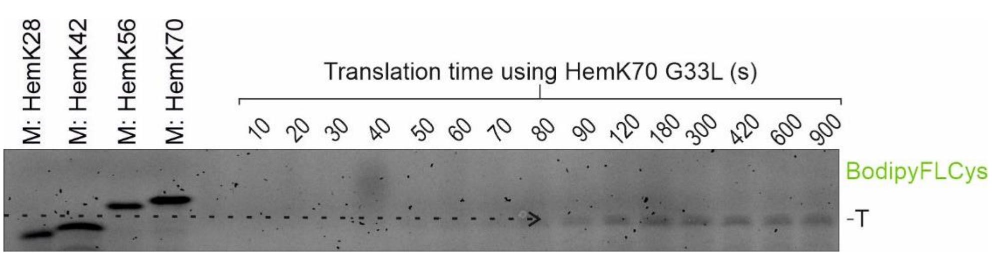

Figure S4. Electrophoretic mobility of the T fragment. Comparison of molecular weight HemK markers labeled N-terminally with BodipyFL (numbers indicate peptide length in aa) with the T fragment obtained by translation of HemK70 G33L construct in the presence of BodipyFLCys-tRNA^{Cys}. Dashed line is a visual guide for the comparison.

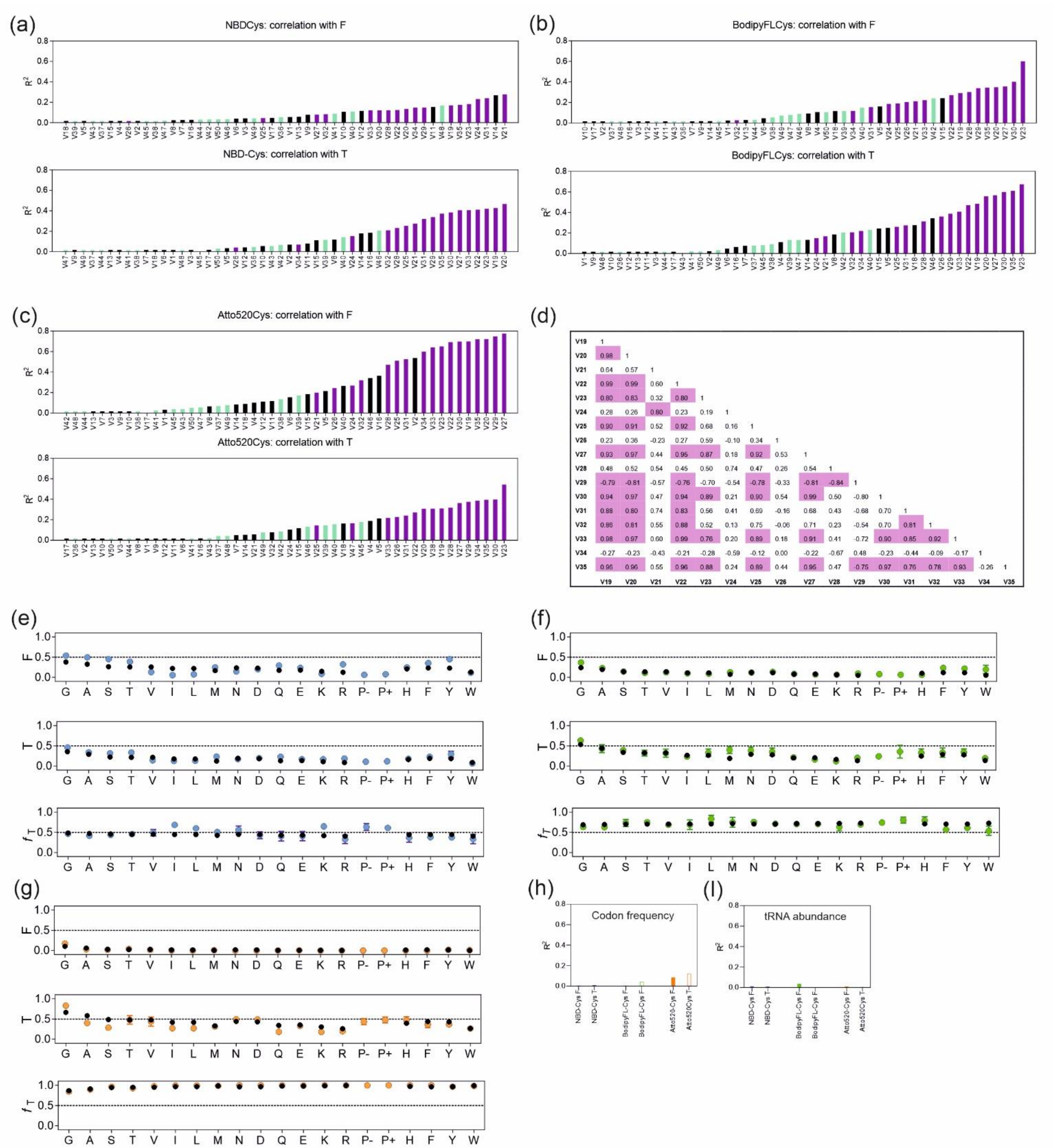

Figure S5. LFER for F and T formation based on properties of the X33 residue.

(a)-(c). Ranking of different regression models based on R^2 for linear correlations of the logarithm of the incorporation levels with the aa descriptors in Table S1. Hydrophobic, steric, and electronic descriptors are shown in black, magenta, and green, respectively. The analysis for the incorporation of (a) NBD, (b) BodipyFL, and (c) Atto520.

(d) Correlation matrix for the steric descriptors with correlations greater than 0.75 highlighted in pink.

(e)-(g) Comparison between the experimental incorporation levels for NBDCys (blue) and values calculated from the coefficients obtained by linear correlation with the length of the amino acid side chain V23 from Table S2 (black). Comparison of the F (top) and T (middle) yields as well as of the f_T value (bottom). (e) NBD. (b) BodipyFL. (c) Atto520.

(h) R^2 values from linear correlations of the logarithm of the incorporation levels with the *E. coli* codon usage frequencies.

(i) R^2 values from linear correlations of the logarithm of the incorporation levels with the *E. coli* tRNA abundance.

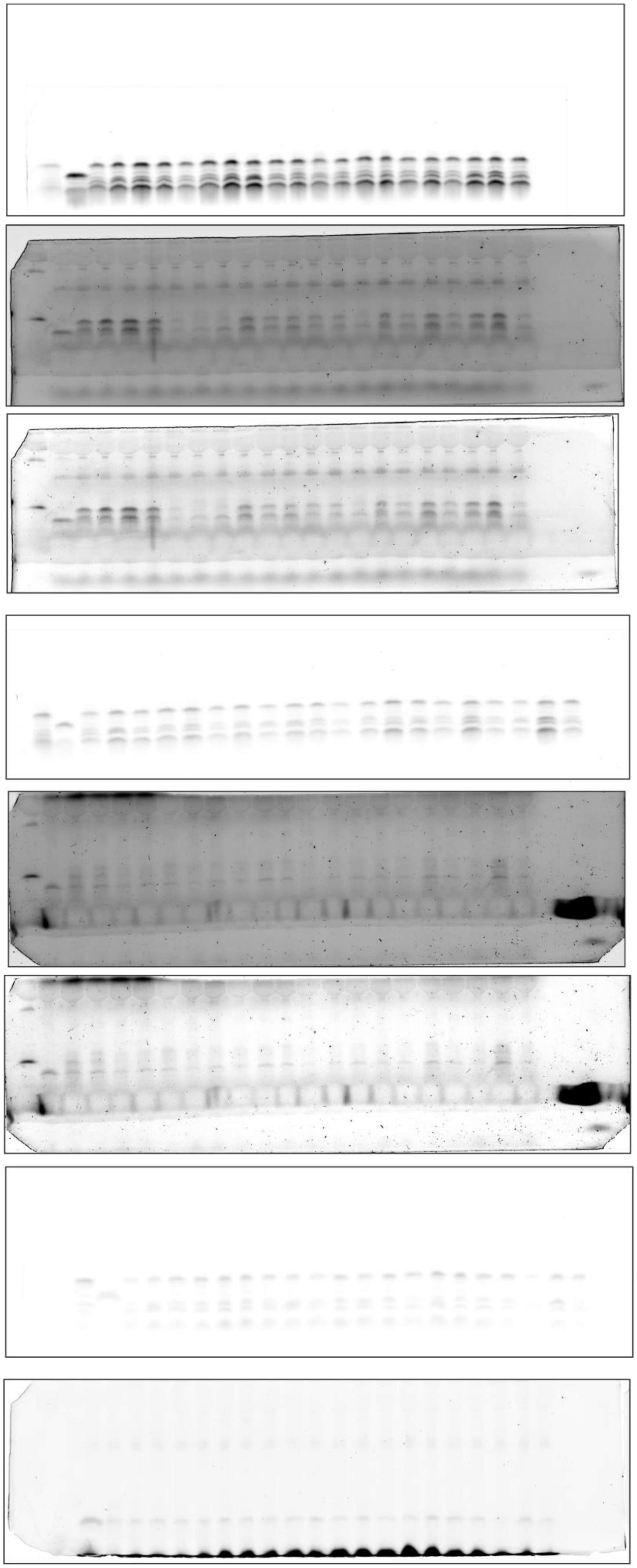

Figure 2a
Atto655

Figure 2a
NBD-Cys

Figure 2a
NBD-Cys
low contrast

Figure 2b
Atto655

Figure 2b
BodipyFL-Cys

Figure 2b
BodipyFL-Cys
low contrast

Figure 2c
Atto655

Figure 2c
Atto520-Cys

Figure S6. Full-size scans of the images in Figure 2.

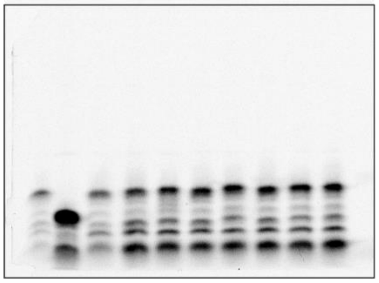

Figure 3a
Atto655

Figure 3a
NBD-Cys

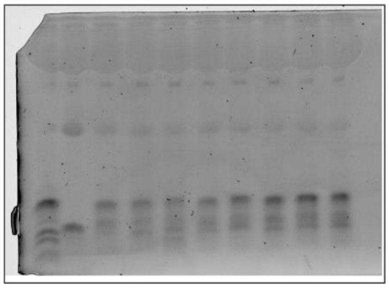

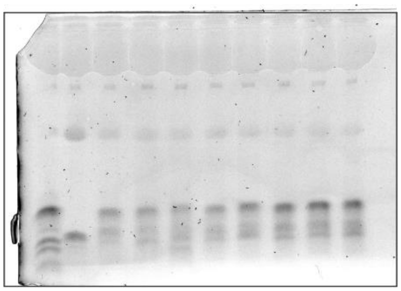

Figure 3a
NBD-Cys
low contrast

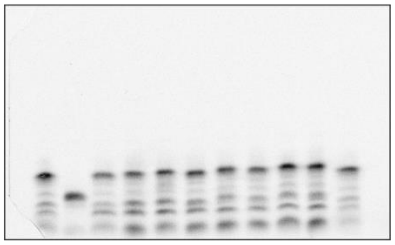

Figure 3b
Atto655

Figure 3b
BodipyFL-Cys

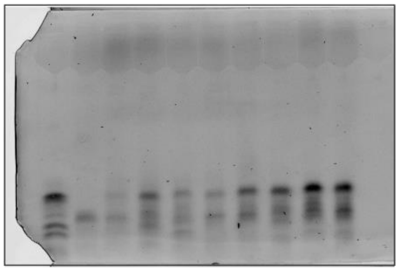

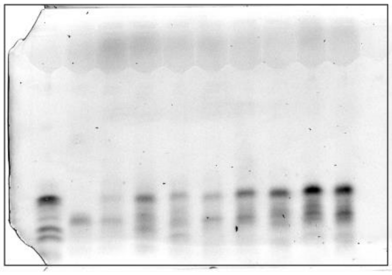

Figure 3b
BodipyFL-Cys
low contrast

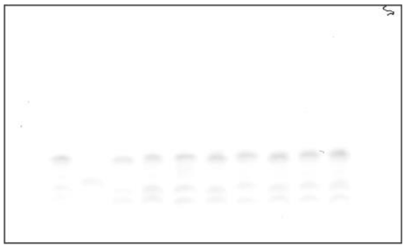

Figure 3c
Atto655

Figure 3c
Atto520-Cys

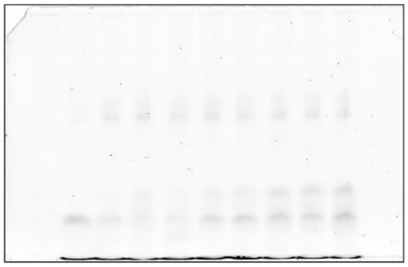

Figure S7. Full-size scans of the images in Figure 3.

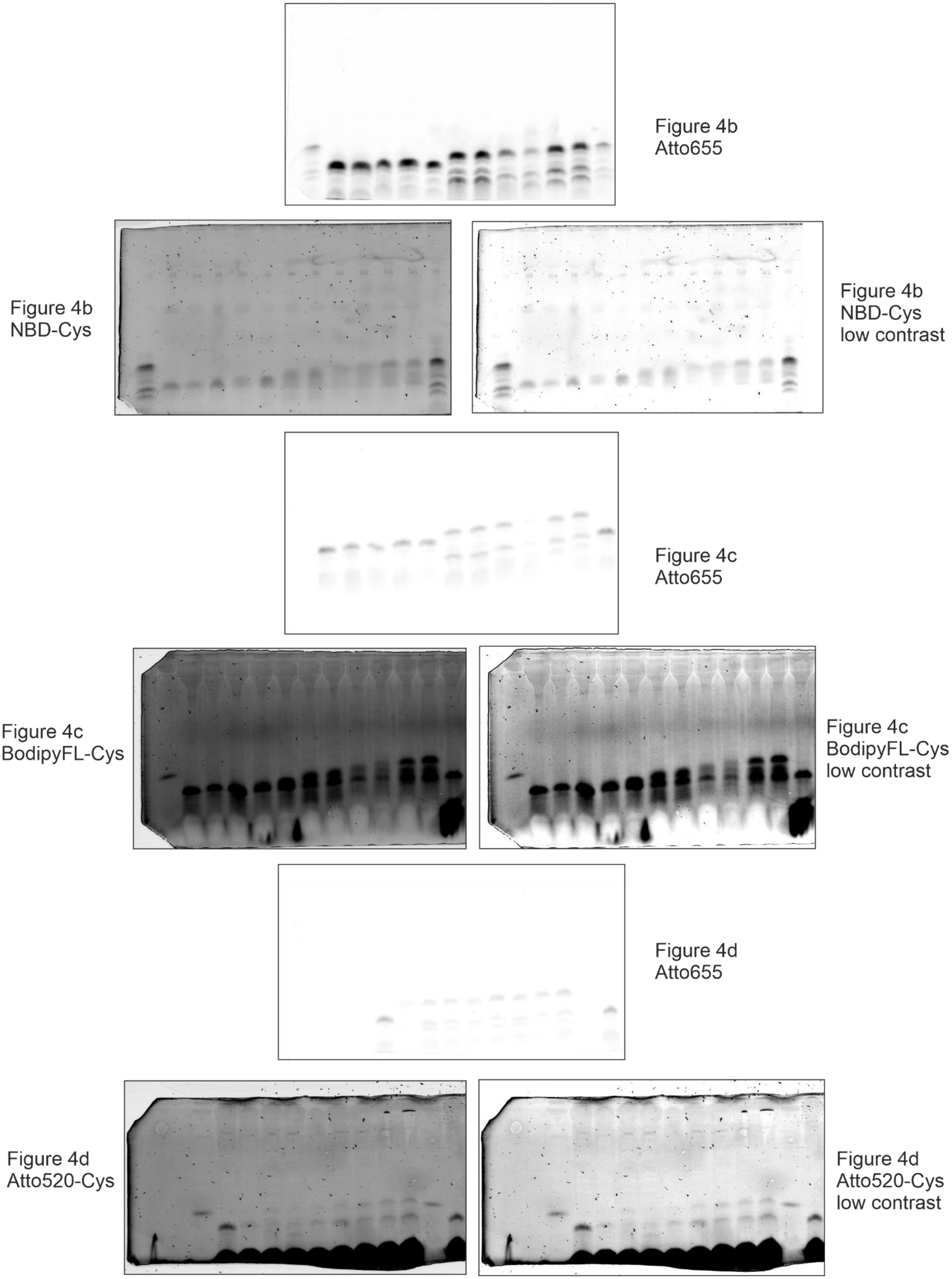

Figure S8. Full-size scans of the images in Figure 4.

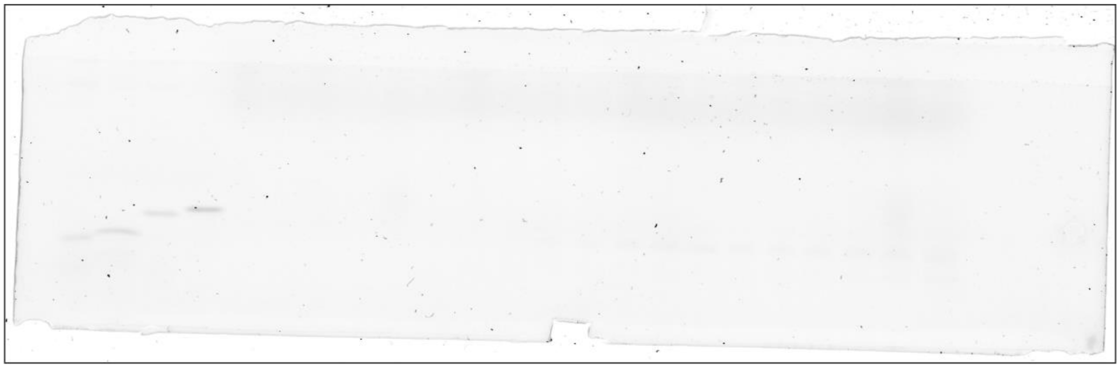

Figure S4
BodipyFL-Cys

Figure S9. Full-size scans of the images in Figure S4.

Table S1: Amino acid descriptors. Descriptors not contained in the AAindex database were from (1).

Variable		AAindex ID[a]
	Hydrophobic descriptors	
V1	Retention coefficient in TFA	BROC820101
V2	Free energy of solution in water	CHAM820102
V3	Solvation free energy	EISD860101
V4	Melting point	FASG760102
V5	Number of hydrogen-bond donors	FAUJ880109
V6	Number of full nonbonding orbitals	FAUJ880110
V7	Partition energy	GUYH850101
V8	Hydration number	HOPA770101
V9	Retention coefficient in HPLC pH 7.4	MEEJ800101
V10	Retention coefficient in HPLC pH 2.1	MEEJ800102
V11	Partition coefficient in thin-layer chromatography	PLIV810101
V12	Retention coefficient at pH 2	GUOD860101
V13	Rf for 1-N-(4-nitrobenzofurazono)-amino acids in ethyl acetate/pyridine/water	[a]
V14	dG of tranfer from organic solvent to water	[a]
V15	Hydration potential or free energy of tranfer from vapor phase to water	[a]
V16	Rf salt chromatoghaphy	[a]
V17	Log D partition coeff. at pH 7.1 for acetylamide derivatives of aa in octanol/H_2O [a]	
V18	dG = fraction buried/accessible amino acids in 22 proteins	[a]
	Steric descriptors	
V19	Average volume of buried residue	CHOC750101
V20	Residue accessible surface area in tripeptide	CHOC760101
V21	Graph shape index	FAUJ880101
V22	Normalized van der Waals volume	FAUJ880103
V23	STERIMOL length of the side chain	FAUJ880104
V24	STERIMOL minimum width of the side chain	FAUJ880105
V25	STERIMOL maximum width of the side chain	FAUJ880106
V26	Average accessible surface area	JANJ780101
V27	Distance between Cα and centroid of side chan	LEVM760102
V28	Side-chain angle θ	LEVM760103
V29	side chain torsion angle φ	LEVM760104
V30	Radius of gyration of side chain	LEVM760105
V31	van der Waals parameter R0	LEVM760106
V32	van der Waals parameter epsilon	LEVM760107
V33	Refractivity	MCMT640101
V34	Value of θ (I)	RACS820113
V35	Substituent van der Waals volume	[a]
	Electronic descriptors	
V36	αCH chemical shifts	ANDN920101
V37	αNH chemical shifts	BUNA790101
V38	A parameter of charge transfer capability	CHAM830107
V39	A parameter of charge transfer donor capability	CHAM830108
V40	Nuclear magnetic resonance (NMR) chemical shift of α-carbon	FAUJ880107
V41	Localized electrical effect	FAUJ880108
V42	Positive charge	FAUJ880111

V43	Negative charge	FAUJ880112
V44	Polarity	GRAR740102
V45	Net charge	KLEP840101
V46	Amphiphilicity index	MITS020101
V47	Isoelectric point	ZIMJ680104
V48	Electron-ion interaction potential values	COSI940101
V49	pKaNH2(NH2 on C-α)	FASG760104
V50	pKaCOOH(COOH on C-α)	FASG760105

[a] https://www.genome.jp/aaindex/

Table S2: Coefficients from the linear regression of log(F) and log(T) with steric descriptors

	NBD			BodipyFL			Atto520		
	b_T	b_F	b_F/b_T	b_T	b_F	b_F/b_T	b_T	b_F	b_F/b_T
V19	-0.004 ± 0.001	-0.003 ± 0.001	0.83	-0.003 ± 0.001	-0.003 ± 0.001	1.02	-0.002 ± 0.001	-0.011 ± 0.002	4.65
V20	-0.004 ± 0.001	-0.003 ± 0.001	0.78	-0.003 ± 0.001	-0.003 ± 0.001	1.03	-0.002 ± 0.001	-0.011 ± 0.002	4.82
V21	-0.13 ± 0.05	-0.15 ± 0.06	1.11	0.07 ± 0.04	-0.10 ± 0.05	1.47	-0.04 ± 0.04	-0.2 ± 0.1	4.04
V22	-0.08 ± 0.02	-0.06 ± 0.03	0.76	-0.06 ± 0.02	-0.06 ± 0.02	0.99	-0.05 ± 0.02	-0.27 ± 0.06	5.66
V23	-0.11 ± 0.03	-0.10 ± 0.04	0.93	-0.10 ± 0.02	-0.12 ± 0.02	1.24	-0.09 ± 0.02	-0.28 ± 0.07	3.14
V24	-0.5 ± 0.2	-0.8 ± 0.3	1.39	-0.4 ± 0.2	-0.5 ± 0.3	1.46	-0.3 ± 0.2	-1.0 ± 0.6	3.15
V25	-0.08 ± 0.03	-0.05 ± 0.04	0.64	-0.06 ± 0.02	-0.06 ± 0.03	1.12	-0.04 ± 0.03	-0.3 ± 0.1	7.11
V26	-0.001 ± 0.003	-0.002 ± 0.003	1.43	-0.004 ± 0.001	-0.004 ± 0.002	0.98	-0.004 ± 0.002	-0.014 ± 0.008	3.81
V27	-0.16 ± 0.05	-0.11 ± 0.07	0.73	-0.14 ± 0.03	-0.14 ± 0.05	1.03	-0.11 ± 0.04	-0.5 ± 0.1	4.61
V28	-0.004 ± 0.002	-0.004 ± 0.002	0.94	-0.004 ± 0.001	-0.005 ± 0.002	1.28	-0.004 ± 0.001	-0.009 ± 0.003	2.43
V29	0.006 ± 0.002	0.005 ± 0.003	0.86	0.005 ± 0.001	0.006 ± 0.002	1.22	0.005 ± 0.002	0.015 ± 0.003	3.09
V30	-0.3 ± 0.1	-0.3 ± 0.1	0.85	-0.28 ± 0.06	-0.30 ± 0.09	1.07	-0.24 ± 0.07	-0.9 ± 0.2	4.01
V31	-0.16 ± 0.05	-0.15 ± 0.06	0.99	-0.10 ± 0.04	-0.09 ± 0.06	0.98	-0.09 ± 0.04	-0.4 ± 0.1	3.87
V32	-0.9 ± 0.4	-0.7 ± 0.5	0.71	-0.6 ± 0.3	-0.3 ± 0.4	0.47	-0.4 ± 0.3	-4.0 ± 2.0	11.08
V33	-0.016 ± 0.004	-0.012 ± 0.006	0.73	-0.011 ± 0.003	-0.011 ± 0.005	0.97	-0.008 ± 0.004	-0.05 ± 0.01	6.45
V34	0.009 ± 0.009	0.017 ± 0.010	1.84	0.01 ± 0.01	0.01 ± 0.01	0.96	0.017 ± 0.005	0.05 ± 0.02	3.11
V35	-0.008 ± 0.002	-0.007 ± 0.003	0.93	-0.007 ± 0.001	-0.007 ± 0.002	0.98	-0.006 ± 0.002	-0.027 ± 0.006	4.73

Table S3: List of primer for construct design

Name	Sequence 5'-3'
HemK_G33A_K34C_m_F	CTG CTG GAA CAT GTT ACC GCA TGC GGG CGT ACT TTT ATC
HemK_G33A_K34C_m_R	GAT AAA AGT ACG CCC GCA TGC GGT AAC ATG TTC CAG CAG
HemK_G33D_K34C_m_F	CTG CTG GAA CAT GTT ACC GAC TGC GGG CGT ACT TTT ATC
HemK_G33D_K34C_m_R	GAT AAA AGT ACG CCC GCA GTC GGT AAC ATG TTC CAG CAG
HemK_G33E_K34C_m_F	CTG CTG GAA CAT GTT ACC GAA TGC GGG CGT ACT TTT ATC
HemK_G33E_K34C_m_R	GAT AAA AGT ACG CCC GCA TTC GGT AAC ATG TTC CAG CAG
HemK_G33F_K34C_m_F	GCT GGA ACA TGT TAC CTT CTG CGG GCG TAC TTT TAT C
HemK_G33F_K34C_m_R	GAT AAA AGT ACG CCC GCA GAA GGT AAC ATG TTC CAG C
HemK_G33H_K34C_m_F	CTG CTG GAA CAT GTT ACC CAC TGC GGG CGT ACT TTT ATC
HemK_G33H_K34C_m_R	GAT AAA AGT ACG CCC GCA GTG GGT AAC ATG TTC CAG CAG
HemK_G33I_K34C_m_F	CTG CTG GAA CAT GTT ACC ATC TGC GGG CGT ACT TTT ATC
HemK_G33I_K34C_m_R	GAT AAA AGT ACG CCC GCA GAT GGT AAC ATG TTC CAG CAG
HemK_G33K_K34C_m_F	CTG CTG GAA CAT GTT ACC AAA TGC GGG CGT ACT TTT ATC
HemK_G33K_K34C_m_R	GAT AAA AGT ACG CCC GCA TTT GGT AAC ATG TTC CAG CAG
HemK_G33L_K34C_m_F	CTG CTG GAA CAT GTT ACC CTG TGC GGG CGT ACT TTT ATC
HemK_G33L_K34C_m_R	GAT AAA AGT ACG CCC GCA CAG GGT AAC ATG TTC CAG CAG
HemK_G33M_K34C_m_F	CTG CTG GAA CAT GTT ACC ATG TGC GGG CGT ACT TTT ATC
HemK_G33M_K34C_m_R	GAT AAA AGT ACG CCC GCA CAT GGT AAC ATG TTC CAG CAG
HemK_G33N_K34C_m_F	CTG CTG GAA CAT GTT ACC AAC TGC GGG CGT ACT TTT ATC
HemK_G33N_K34C_m_R	GAT AAA AGT ACG CCC GCA GTT GGT AAC ATG TTC CAG CAG
HemK_G33P_K34C_m_F	GCT GGA ACA TAGT TAC CCC GTG CGG GCG TAC TTT TAT C
HemK_G33P_K34C_m_R	GAT AAA AGT ACG CCC GCA CGG GGT AAC ATG TTC CAG C
HemK_G33Q_K34C_m_F	CTG CTG GAA CAT GTT ACC CAG TGC GGG CGT ACT TTT ATC
HemK_G33Q_K34C_m_R	GAT AAA AGT ACG CCC GCA CTG GGT AAC ATG TTC CAG CAG
HemK_G33R_K34C_m_F	CTG CTG GAA CAT GTT ACC CGT TGC GGG CGT ACT TTT ATC
HemK_G33R_K34C_m_R	GAT AAA AGT ACG CCC GCA ACG GGT AAC ATG TTC CAG CAG
HemK_G33S_K34C_m_F	CTG CTG GAA CAT GTT ACC AGC TGC GGG CGT ACT TTT ATC
HemK_G33S_K34C_m_R	GAT AAA AGT ACG CCC GCA GCT GGT AAC ATG TTC CAG CAG
HemK_G33T_K34C_m_F	CTG CTG GAA CAT GTT ACC ACC TGC GGG CGT ACT TTT ATC
HemK_G33T_K34C_m_R	GAT AAA AGT ACG CCC GCA GGT GGT AAC ATG TTC CAG CAG
HemK_G33V_K34C_m_F	CTG CTG GAA CAT GTT ACC GTA TGC GGG CGT ACT TTT ATC
HemK_G33V_K34C_m_R	GAT AAA AGT ACG CCC GCA TAC GGT AAC ATG TTC CAG CAG
HemK_G33W_K34C_m_F	CTG CTG GAA CAT GTT ACC TGG TGC GGG CGT ACT TTT ATC
HemK_G33W_K34C_m_R	GAT AAA AGT ACG CCC GCA CCA GGT AAC ATG TTC CAG CAG
HemK_G33Y_K34C_m_F	CTG CTG GAA CAT GTT ACC TAC TGC GGG CGT ACT TTT ATC
HemK_G33Y_K34C_m_R	GAT AAA AGT ACG CCC GCA GTA GGT AAC ATG TTC CAG CAG
HemK_GGGG_K34C_m_F	CTG GAA GGT GGC GGT GGC TGC GGG CGT ACT TTT ATC C
HemK_GGGG_K34C_m_R	CCC GCA GCC ACC GCC ACC TTC CAG CAG GAT TTC AGC
HemK_GGTG_K34C_m_F	CTG GAA GGT GGC ACC GGC TGC GGG CGT ACT TTT ATC C
HemK_GGTG_K34C_m_R	CCC GCA GCC GGT GCC ACC TTC CAG CAG GAT TTC AGC
HemK_GVGG_K34C_m_F	CTG GAA GGT GTT GGT GGC TGC GGG CGT ACT TTT ATC C
HemK_GVGG_K34C_m_R	CCC GCA GCC ACC AAC ACC TTC CAG CAG GAT TTC AGC
HemK_GVTG_K34C_m_F	CTG GAA GGT GTT ACC GGC TGC GGG CGT ACT TTT ATC C
HemK_GVTG_K34C_m_R	CCC GCA GCC GGT AAC ACC TTC CAG CAG GAT TTC AGC
HemK_HGGG_K34C_m_F	CTG GAA CAT GGT GGT GGC TGC GGG CGT ACT TTT ATC C
HemK_HGGG_K34C_m_R	CCC GCA GCC ACC ACC ATG TTC CAG CAG GAT TTC AGC

HemK_HGTG_K34C_m_F	CTG GAA CAT GGT ACC GGC TGC GGG CGT ACT TTT ATC C
HemK_HGTG_K34C_m_R	CCC GCA GCC GGT ACC ATG TTC CAG CAG GAT TTC AGC
HemK_HVGG_K34C_m_F	CTG GAA CAT GTT GGT GGC TGC GGG CGT ACT TTT ATC
HemK_HVGG_K34C_m_R	GAT AAA AGT ACG CCC GCA GCC ACC AAC ATG TTC CAG

SUPPLEMENTAL REFERENCES

1. Mei, H.; Liao, Z. H.; Zhou, Y.; Li, S. Z., A new set of amino acid descriptors and its application in peptide QSARs. *Biopolymers* **2005,** *80* (6), 775-86.
